# Expression and Characterization of Purinergic Receptors in Rat Middle Meningeal Artery–Potential Role in Migraine

**DOI:** 10.1371/journal.pone.0108782

**Published:** 2014-09-29

**Authors:** Kristian Agmund Haanes, Lars Edvinsson

**Affiliations:** Department of Clinical Experimental Research, Copenhagen University Hospital, Glostrup, Denmark; Thomas Jefferson University, United States of America

## Abstract

The dura mater and its vasculature have for decades been central in the hypothesis of migraine and headache pathophysiology. Although recent studies have questioned the role of the vasculature as the primary cause, dural vessel physiology is still relevant in understanding the complex pathophysiology of migraine. The aim of the present study was to isolate the middle meningeal artery (MMA) from rodents and characterize their purinergic receptors using a sensitive wire myograph method and RT-PCR. The data presented herein suggest that blood flow through the MMA is, at least in part, regulated by purinergic receptors. P2X1 and P2Y6 receptors are the strongest contractile receptors and, surprisingly, ADPβS caused contraction most likely via P2Y1 or P2Y13 receptors, which is not observed in other arteries. Adenosine addition, however, caused relaxation of the MMA. The adenosine relaxation could be inhibited by SCH58261 (A2A receptor antagonist) and caffeine (adenosine receptor antagonist). This gives one putative molecular mechanism for the effect of caffeine, often used as an adjuvant remedy of cranial pain. Semi-quantitative RT-PCR expression data for the receptors correlate well with the functional findings. Together these observations could be used as targets for future understanding of the *in vivo* role of purinergic receptors in the MMA.

## Introduction

The dura mater and its vasculature have for decades been central to many hypotheses aimed to explain migraine and headache pathophysiology. Already in the 40 s it was shown that direct stimulation on the dura and its vasculature, caused a headache resembling that of migraine [1]. Previous and today's treatments for acute migraine are vasoconstrictors such as triptans [2]. In recent years, however, there are results challenging the importance of only dilatation of dural vessels as the primary mechanism behind migraine, for example not all MMA (Middle Meningeal Artery) vasodilators cause migraine-like attacks e.g. acetylcholine/carbachol and VIP (Vasoactive Intestinal Peptide) [3], [4]. Recent MRI studies did not reveal any dilatation during an acute migraine attack, however the authors could not exclude possible dilatation of dural branches of the MMA as the intracranial MMA diameter was not measured [5]. Even if dilatation of the dural vessels could be a side phenomenon, the dural vessels may play an important role in migraine pathophysiology [6]. Consequently, possible vasomotor receptor agonists or antagonists could have therapeutic potential, at least in relation to the cranial pain in migraine attacks.

The purinergic system is a complex signalling system and there exist today 19 receptors in total (18 in rodents) [7]. Purinergic receptors are divided into two main groups: the adenine nucleoside receptors (A/P1) and the purine/pyrimidine nucleotide receptors (P2). The P2 receptors are further divided into P2X receptors that are ligand-gated ion channels and P2Y receptors that are G-protein coupled receptors [7]. The system is made further complex by findings that nucleotide/side breakdown enzymes may interconvert the different agonists [8]. Therefore, non-degradable analogues are now of great assistance when one embarks on a purinergic receptor characterization [9], [10].

The role of the purinergic system has not before been evaluated in depth in relation to the dura mater and the meningeal vasculature. The extracellular nucleosides (e.g. adenosine) and nucleotides (e.g. ATP/UTP) act on purinergic receptors, regulating contraction and relaxation of blood vessels [11], [12]. Despite the importance of contractile/relaxing receptors, to this date there is no study of purinergic receptors in the MMA. This might be due to the technical difficulty of performing myograph analysis in rodents. Purinergic receptors have however, been investigated in relation to migraine, where there is a clear involvement of P2X3 in the trigeminal ganglion [13], [14], and afferents supplying the dura, express P2X3 receptors [15].

Interestingly, caffeine is an antagonist of adenosine receptors and is probably the most consumed antagonist in the world. The presence of caffeine in many painkillers and the positive effect of caffeine on hangover headache illustrates that caffeine is an effective remedy for headache [16], [17]. Caffeine can have direct analgesic effects by elevating nociceptive thresholds [18]. However, during caffeine withdrawal (the absence of caffeine after long time consumption) a headache can often be observed, suggesting that lack of caffeine is inducing the headache. Since cranial pain is the symptom of caffeine removal, there seems to be a special relation between caffeine and headache, and there seems to be consensus that vasoconstrictor actions of caffeine are implicated in relief of headache [17], [18]. One of the proposed mechanisms for the caffeine effects is a blockade of the adenosine receptors; particularly the Gs coupled A2A and A2B receptors leading to a reduced relaxation of the blood vessel [17], [19], [20]. However, this has never been studied on dural blood vessels [21].

The present study was designed to investigate the vasomotor effects of purinergic receptors in the MMA, using functional myograph studies with natural and designed agonists. For this purpose we have developed a method to isolate and perform wire myograph experiments on the MMA of rat, with high reproducibility. Here, we present the pharmacological characteristics of several purinergic receptors present in the MMA and assist this with PCR demonstrations. Caffeine was found to reverse the relaxing effect of adenosine, suggesting one possible mechanism for the reduced sensation of cranial pain associated with caffeine intake. Together this provides a novel insight into putative involvement of purinergic receptors mechanisms in the MMA.

## Methods

### MMA isolation

**Figure 1:**
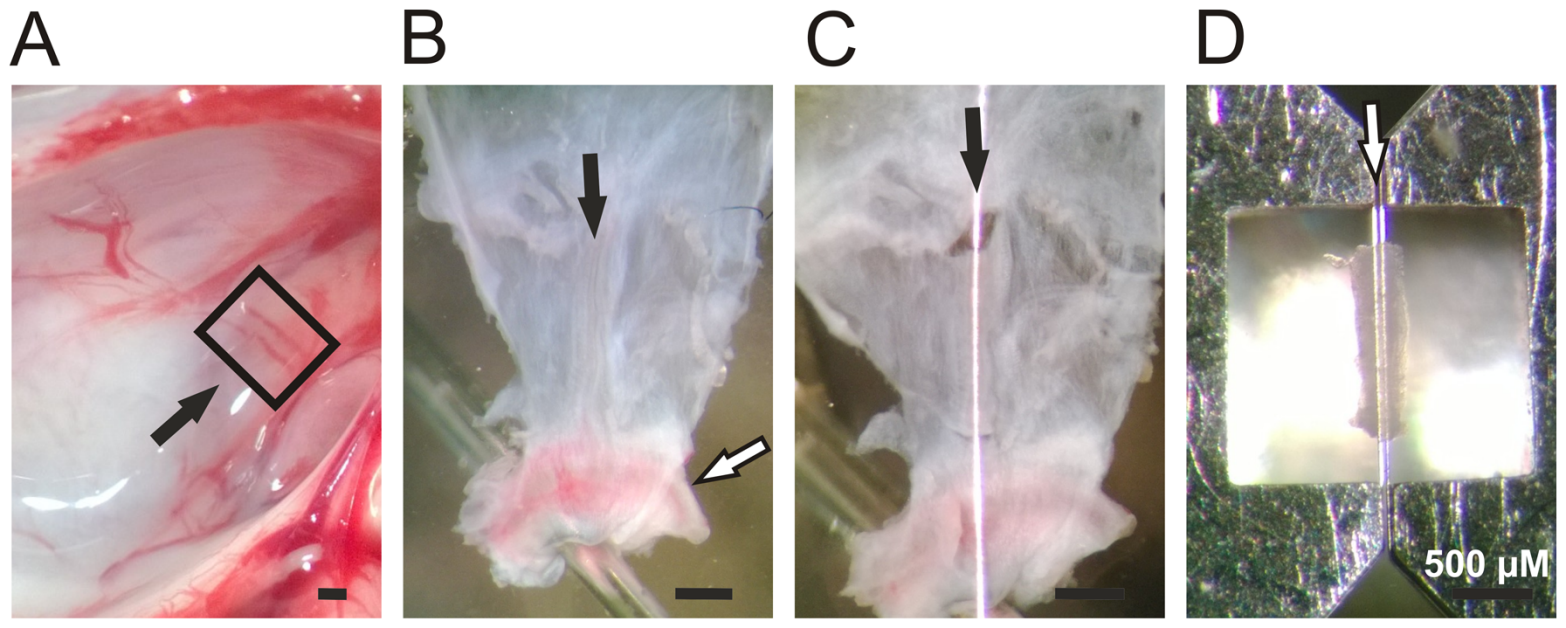
Isolation of the middle meningeal artery (MMA). A) The MMA on the inside of the rat scull, the arrow illustrates which fragment was utilised. B) The dura is dissected, stretched and held by needles, the MMA is shown with the black arrow and the internal maxillary artery (IMA) with the white arrow. C) A 25 µm steel wire (arrow) is guided through the MMA after it has been cut into a ∼2 mm segment. D) The MMA (here ∼1.350 mm long) is mounted with a second wire, in a wire myograph (arrow). Scale bars are 500 µm.

MMAs were isolated from male Sprague-Dawley rats (n = 24, 250 g), which were decapitated under CO_2_ anaesthesia. Procedures are approved by the Danish Animal Experiment Inspectorate (Dyreforsøgstilsynet). The cranium was carefully surgically opened, brain removed and a 5×10 mm piece of the dura surrounding the MMA dissected free using a dissection microscope, including the first part of the internal maxillary artery (IMA). The dura mater segment was stretched and held by needles in a Petri dish (Fig. 1) in Ca^2+^ free KREBs containing NaCl 119 mM, NaHCO_3_ 15 mM, KCl 4.6 mM, MgCl_2_ 1.2 mM, NaH_2_PO_4_ 1.2 mM, 5.5 mM glucose and 30 µM EDTA. After the dissection, NaKREBs buffer with similar composition but including 1.5 mM CaCl_2_ and no EDTA was used. The segment closest to the IMA, before branching, was used for myograph studies (o.d. ∼100 µm). The endothelial cells were removed with the insertion of the first myograph wire. All solutions where aerated with gas composed of 95% O_2_ and 5% CO_2_, to maintain a pH of 7.4.

### Myography

Each ∼2 mm long segment of the MMA was mounted in an arterial myograph, on a pair of 25 µm metal wires (Fig. 1). One wire was connected to a micrometer screw where vascular circumference can be adjusted and the other to a force displacement transducer, paired with an analogue/digital converter (ADInstruments, Oxford, UK). Data were recorded on a computer using a PowerLab unit and LabChart (ADInstruments) was used for recording and calculations.

All experiments were conducted at +37°C. The segments were normalised to 90% of the internal circumference a vessel would have at 50 mmHg. In normal blood vessels, the pressure (e.g. 100 mmHg) will be distributed evenly around the vessel wall, with the two wires and high point pressure, 50 mmHg was considered optimal for contraction curves. A reference value for each segment was created by replacing part of the NaCl with KCl (60 mM K^+^). Segments with maximum contractile capacity of less than 0.1 mN were excluded. Segments were either subjected to the agonists in NaKREBs or stably pre-contracted with 30 mM K^+^, in the relaxation experiments. For all substances a cumulative concentration-response curve was made, except for αβmetATP (α,β-Methyladenosine5′-triphosphate) where the curve was made for every second data point and experiments pooled, due to the rapid desensitisation by this particular agonist for P2X receptors. Experiments with antagonists were performed in parallel.

### RNA isolation and PCR

Six MMAs were pooled and homogenised using beads in lysis buffer from RNeasy Mini Kit (Qiagen 74104), and RNA was isolated (n = 3). Briefly, MMA lysates were precipitated with 1∶1 volume of 70% ethanol and column purified. Extracted RNA (5 ng) was used per reaction mixture in QIAGEN OneStep RT-PCR Kit (210212) analysis, with amplification parameters as follows: one cycle at 50°C for 30 min and one cycle at 95°C for 15 min followed by 40 cycles at 95°C for 30 s, 58°C for 30 s, 72°C for 30 s and, finally, one cycle at 72°C for 10 min. Subsequently, all transcripts were subjected to electrophoresis on 1.5% agarose gels. Table 1 shows primers used; these were synthesised by TAG Copenhagen A/S (Denmark). All primers were designed using Primer-BLAST (NCBI) with an expected Tm of 60°C; annealing temperature was selected to be 2°C below this Tm. All primers are designed with one primer spanning the exon splicing site, so that only mRNA can be detected with these primers (for splice info see table 1). For the P2Y14 receptor this was not possible due to missing sequence/structure data and lack of sequence similarity with the known mouse splicing.

**Table 1:** Primers, distribution and intracellular pathways of purinergic receptors.

Receptor	Exon *	bp	Sequence 5 -3	Dst ‡	Ligand	Signal ¤
P2X1	6→7/8	182	CAGCTTTCCACGCTTCAAGG	++	ATP	Depol.-Ca2+↑
NM_012997.3			CAACCACCCCACCCTTCTCA			
P2X2	4→6/7	250	TACCTGCCATTCAGACGACG	+	ATP	Depol.-Ca2+↑
NM_053656.2			GCTTGCAATGTTGCCCTTTG			
P2X3	1/2→3	172	TCTCCTACTTTGTGGGGTGG	−	ATP	Depol.-Ca2+↑
NM_031075.2			ATGACAAAGACAGAGGTGCCC			
P2X4	9→11/12	208	TACGGCATCCGCTTTGACAT	++	ATP	Depol.-Ca2+↑
NM_031594.1			CCGAAAGACCCTGCTCGTAG			
P2X5	7→8/9	201	CGACTGGGGTCTATTGTCCG	+	ATP	Depol.-Ca2+↑
NM_080780.2			CCTGGCGAACCTGAAGTTGT			
P2X6	9→11/12	242	AATTACTGGTGGGCAGCCTC	−	ATP	Depol.-Ca2+↑
NM_012721.2			GGGGCTCTTGCCTCTTCATA			
P2X7	1→1/2	231	CTTCGGCTACTCTTCGGTGG	(−)	ATP	Depol.-Ca2+↑
NM_019256.1			TCGCTCATCAAAGCAAAGCTAA			
P2Y1	1→1/2	278	ACGACAGGGTTTATGCCACC	+	ADP	Gq-IP3-Ca2+↑
NM_012800.1			AGGGACTTCTTGTGACCATGTTA			
P2Y2	2/3→3	193	CTGATCGGGTCCAGGGCAAT	+	ATP/UTP	Gq-IP3-Ca2+↑
NM_017255.1			GGAAGATGTAGAGGGCCACG			
P2Y4	1/2→2	249	TAAGGAAGCTAGGGGGCCAT	+	UTP	Gq-IP3-Ca2+↑
NM_031680.1			GTCTGACAATGCCAGGTGGA			
P2Y6	2/3→3	160	GCATGAGACAGATTCTCCAAGCA	+++	UDP	Gq-IP3-Ca2+↑
NM_057124.2			CCACCAGCACCACTGAGTAA			
P2Y12	1/2→2	102	AGAGGAAAGCACCAGATGCC	(−)	ADP	Gi - cAMP↓
NM_022800.1			CACCTCCATGGTCCTGGTTA			
P2Y13	1/2→2	96	AAACAAAGCTGATGCTCGGGA	++	ADP	Gi - cAMP↓
§			CAGCTGTGTCATCCGAGTGT			
P2Y14	2	270	TTCTGGATCGTGTTCCTTCTG	+++	UDP-gluc.	Gi - cAMP↓
NM_133577.1			CGAGAGCAGCAGGGTGAATTC			
A1	1→1/2	137	GGCCACAGACCTACTTCCAC	+	Adenosine	Gi - cAMP↓
NM_017155.2			TGTCTTGTACCGGAGAGGGAT			
A2A	2→2/3	184	CCATCCCCTTCGCTATCACC	+++	Adenosine	Gs - cAMP↑
NM_053294.3			AAGCCATTGTACCGGAGTGG			
A2B	1/2→2	147	GCGTCCCGCTCAGGTATAAA	++	Adenosine	Gs - cAMP↑
NM_017161.1			GTTCTGTGCAGTTGCTGGTG			
A3	1/2→2	166	ACAGTCAGATATAGAACGGTTACCA	+++	Adenosine	Gi - cAMP↓
NM_012896.2			AACGGAAGTGGCATGAGAGG			

*Exon splicing: Whole number (e.g 6) primer within the exon. Split numbers (e.g. 7/8), primers overlapping exons.

‡ The distribution pattern, representative of n = 3 RT-PCRs. A weak product in one sample is denoted (−).

§ Mouse (NM_028808.3) was used for primer design and changed for rat (NM_001002853.1 and NW_001084804.1).

¤ Signal pathways, adapted from Burnstock [7].

### Chemicals and analysis

αβmetATP (Cat. #3209) and SCH58261 (Cat. #2270) were purchased from Tocris Bioscience (UK). Thio-phosphates were purchased from Biolog Life Science Institute (Bremen, Germany). All remaining chemicals were purchased from Sigma Aldrich (Germany). Data were analysed using excel and GraphPad Prism, and figures prepared using CorelDraw. The pEC_50_ values are the negative logarithm of the EC_50_ value and were calculated using nonlinear regression. For E_max_ calculations, the maximal value at the highest concentration added was used. Two-way ANOVA with repeated measurements was used to compare concentration–response curves obtained for adenosine with caffeine inhibition. All statistical analyses were considered significant when p<0.05.

## Results

### MMA study technique

**Figure 2:**
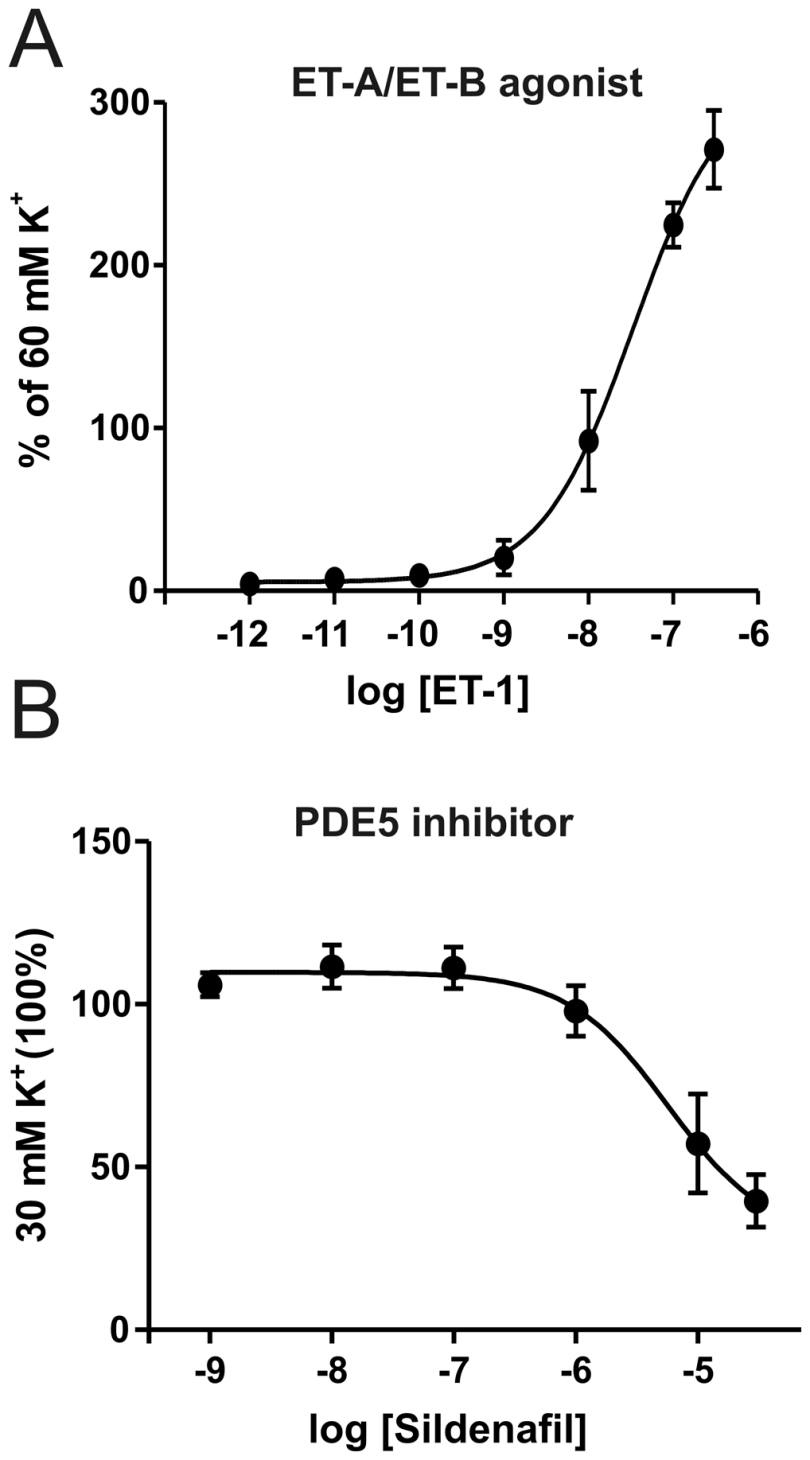
Characterization of responses to a known vasoconstrictor/dilator. A) MMA contraction in response to the ET-A/ET-B receptor agonist ET-1, 100% equals the contraction in response to 60 mM K^+^ B) MMA relaxation in response to sildenafil a PDE5 inhibitor, 100% is set to be the pre-contraction with 30 mM K^+^. (n = 5)

The method used was optimised and the detailed procedure is presented in Fig. 1. The isolated MMAs were mounted and showed an average K^+^ response of 0.5±0.1 mN (n = 20). Endothelial cells were removed with the insertion of the wire. There was no detected endothelium dependent functional response in any of the vessels tested with pre-contraction with 10^−5^ M PGF_2α_ followed by 10^−5^ M carbachol. This allowed a study on only the vascular smooth muscle cells. We also investigated the standard responses in the blood vessels. MMAs contracted strongly when endothelin-1 was added (Fig. 2A). The PDE5 inhibitor, sildenafil (Viagra) caused vasodilatation as expected (Fig. 2B). Together this showed that the dura vessels behaved as expected.

### Pharmacology

#### Contractile responses

**Figure 3:**
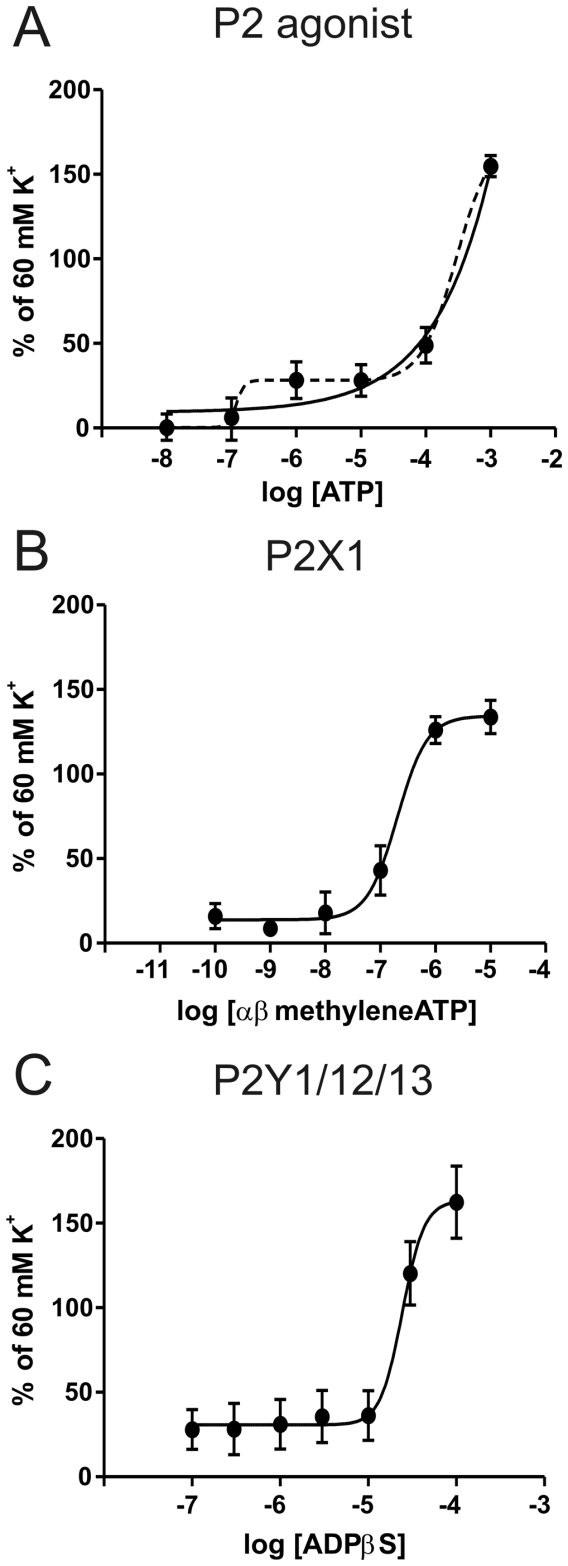
Contractile response to purine nucleotides. A) Response to ATP could be interpreted as either biphasic (stapled line) or sigmoidal (full line). The dose response is made in the presence of SCH580261 (A2A antagonist), to prevent relaxation by the produced adenosine. B) Response to αβmetATP, a P2X1 agonist. The response curve was created using 100 fold increments in the dose response curve to avoid desensitisation. C) Contraction in response to ADPβS, a stable ADP analogue and P2Y1/12/13 agonist. (n = 5)

Our next goal was to investigate what purinergic receptors are functionally expressed in the MMA. Below available agonists were used and rely much on the available nomenclature for defining purinergic receptors [11]. Fig. 3A shows that ATP (in the presence of SCH58261 for A2A receptor inhibition, to prevent adenosine induced relaxation) can be interpreted as a biphasic response with a low and a high EC_50_ or as a sigmoidal response with only a high EC_50_. At low concentrations (10^−8^ to 10^−6^ M) there is a rapid brief contraction. At high concentration there is a strong contractile response (10^−5^ to 10^−3^ M) to ATP. However, ATP is well known to break down fast. In order to circumvent this problem, αβmetATP was used. This agonist elicits a strong concentration-dependent contraction (Fig. 3B) and in the concentrations we used, it is considered to act on the P2X1 receptor (although it could also activate the P2X3 receptor). The P2X1 receptor is the strongest expressed P2X receptor in the vascular system [22].

We further embarked on the analysis of P2Y receptors. The agonist ADPβS, which activates the G-protein coupled purine receptors (P2Y1/12/13), caused a strong contractile response, which is not observed in other arteries (cerebral, omental or mesenteric arteries in rats [9], [10], see discussion).

**Figure 4:**
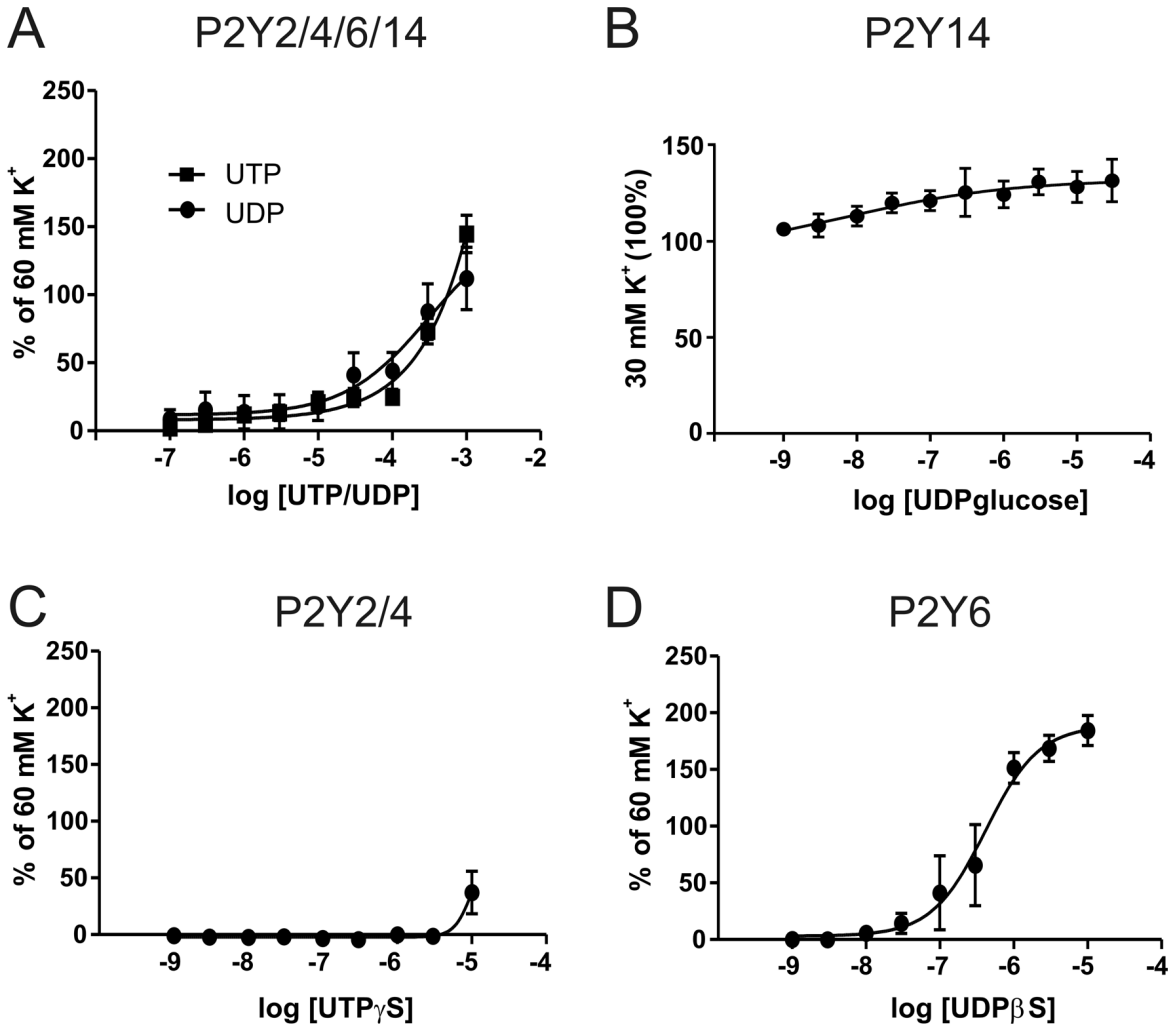
Contractile responses to pyrimidine nucleotides. Contractile responses to UTP (P2Y2/4) and UDP (P2Y6/14) (A), to UDP-glucose a P2Y14 agonist on vessels pre-contracted with 30 mM potassium (B), the non-degradable UTP analogue UTPγS, a specific P2Y2/4 agonist (C) and the non-degradable UDP analogue UDPβS, a specific P2Y6 agonist (D). (n = 5)

The other P2Y receptors are activated by pyrimidines. The P2Y2/4 receptors agonist UTP caused a contraction which was very similar to that of the P2Y6 receptor agonist UDP (Fig. 4A). UDP glucose, recently characterised vasoconstrictor acting on P2Y14 receptor [23], [24], caused a weak contraction (Fig. 4B). Interestingly, when we used the stable UTP and UDP analogues (UTPγS and UDPβS, respectively), it was observed that the P2Y6 receptor is a stronger vasoconstrictor when compared to activation of the P2Y2/4 receptors (Fig. 4C/D). This suggests that UTP is rapidly broken down and the formed UDP can act on the P2Y6 receptor causing contraction.

#### Dilatation responses

**Figure 5:**
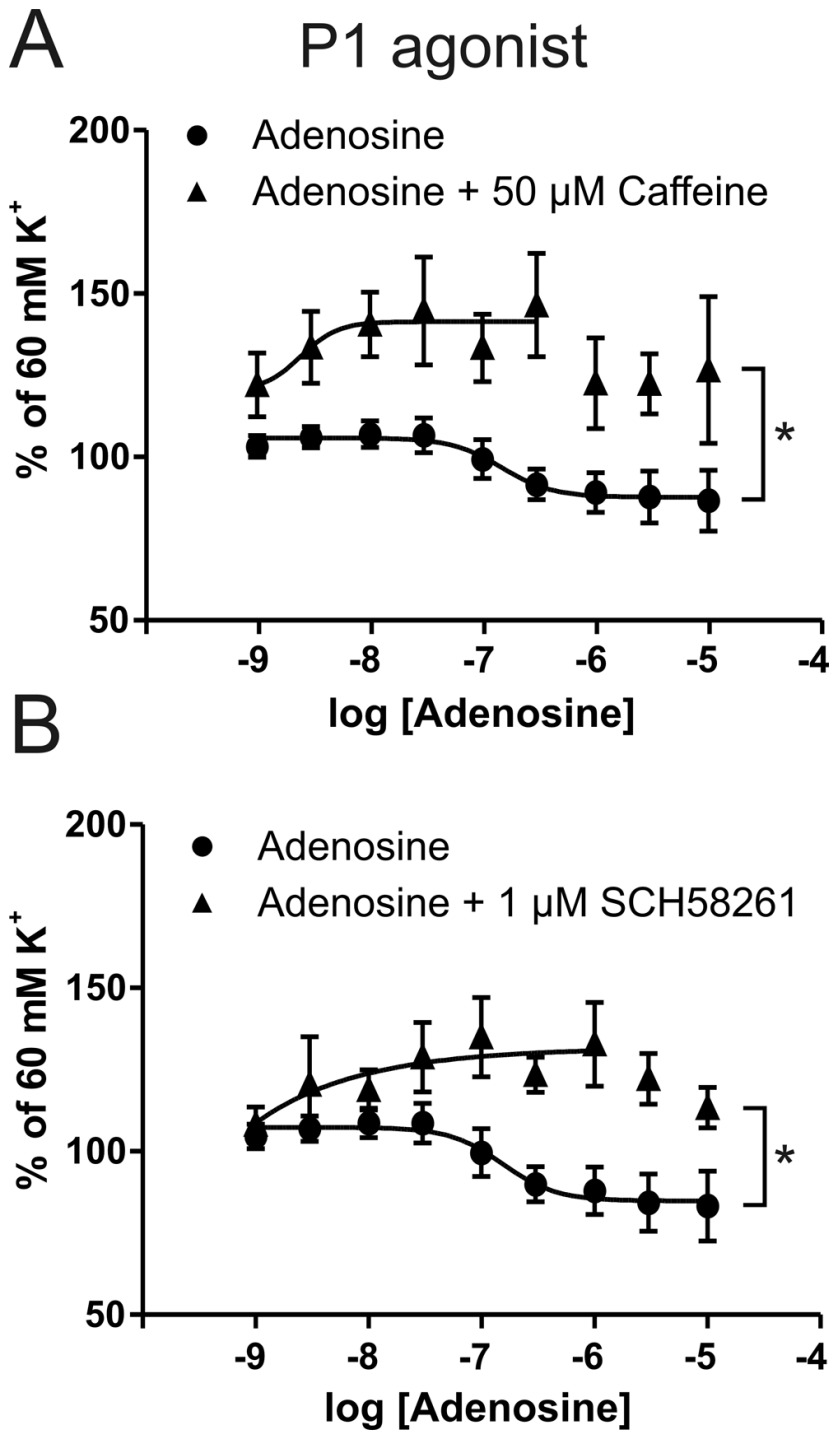
Effects of adenosine and caffeine on MMA. A) Adenosine causes a relaxation of the MMA, and 50 µM caffeine inhibits the relaxation and causes a contraction * = p_curve_ = 0.029, with two way ANOVA. B) 1 µM SCH58261, a specific A2A antagonist mimics the antagonism of caffeine * = p_curve_ = 0.015, with two way ANOVA (n = 5).

In pre-contracted MMA segments, adenosine results in relaxation (Fig. 5A/B). Both the case of caffeine withdrawal headache and that caffeine itself is also a headache reliever, presumably working on adenosine receptors [16], [17]. Here we report that caffeine indeed not only blocks the relaxing effect of adenosine, but caused adenosine to elicit a contraction (Fig. 5A). The response to caffeine can in part be mimicked by the A2A receptor antagonist SCH58261 (Fig. 5B), which is potent and highly selective [25].

#### Overall pharmacology responses

**Figure 6:**
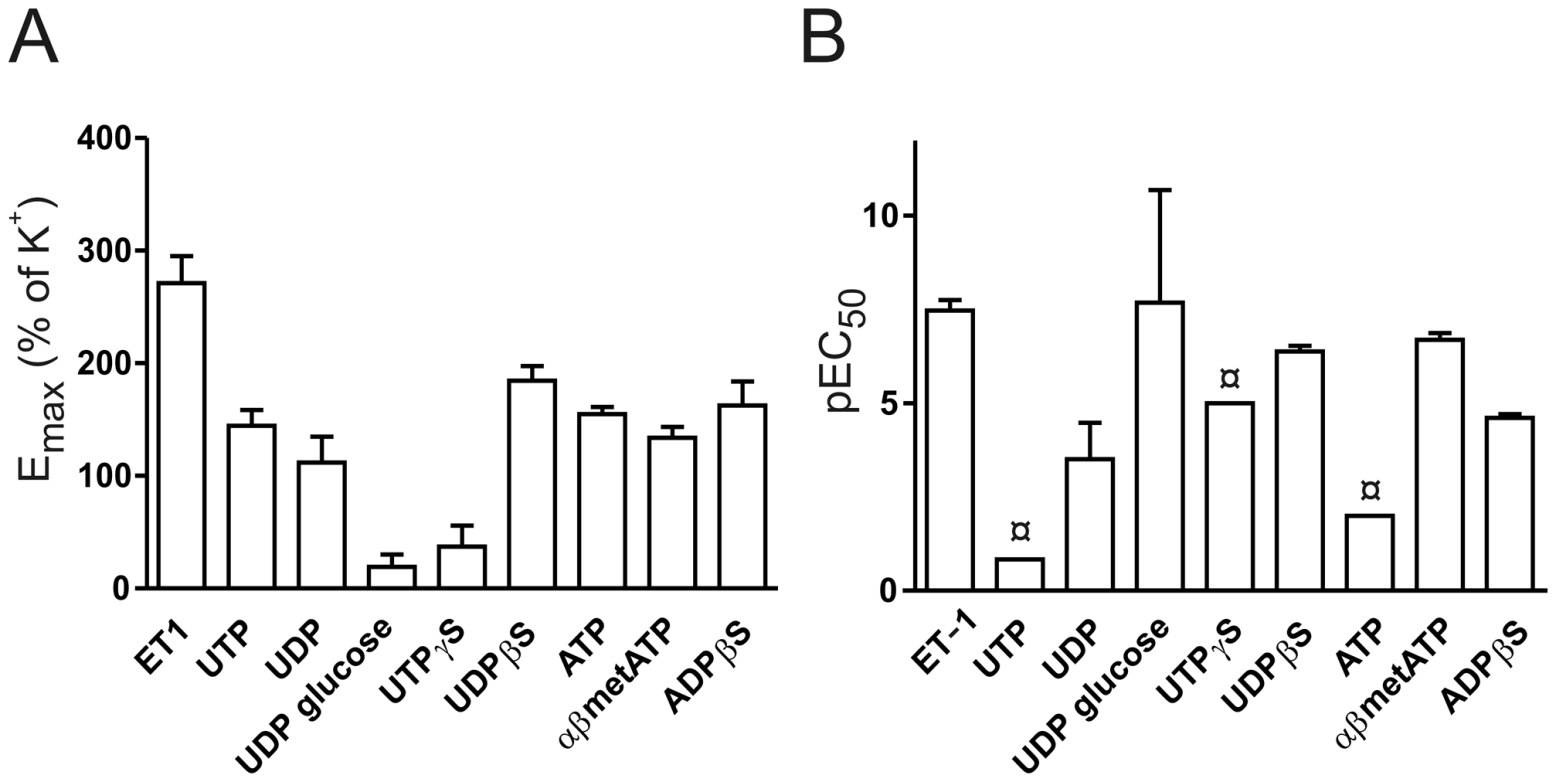
E_max_ and pEC_50_ of the purinergic agonists. A) E_max_ values are presented and are the max value obtained at maximum concentration used in the above experiments. The E_max_ is therefore not the absolute max concentration. B) pEC_50_ values, calculated using non-linear regression in graph pad. For the curves with no clear E_max_ and therefore a large STD, pEC_50_ values are only estimates and are denoted with ¤.

For the purinergic responses observed in the MMA we made calculations of the pEC_50_ (Fig. 6A) and E_max_ values (Fig. 6B) induced by the different agonists. The results show that UDPβS, αβmetATP and ADPβS were the strongest and most potent contracting stimulants. They have a high pEC_50_ and a high E_max_. This suggests that P2X1, P2Y6 and P2Y1/12/13 receptors are the functionally most important contractile purinergic receptors in the rat MMA. For the dilatation responses, the A2A receptor seems to be the strongest adenosine receptor.

### Molecular biology

#### mRNA findings

**Figure 7:**
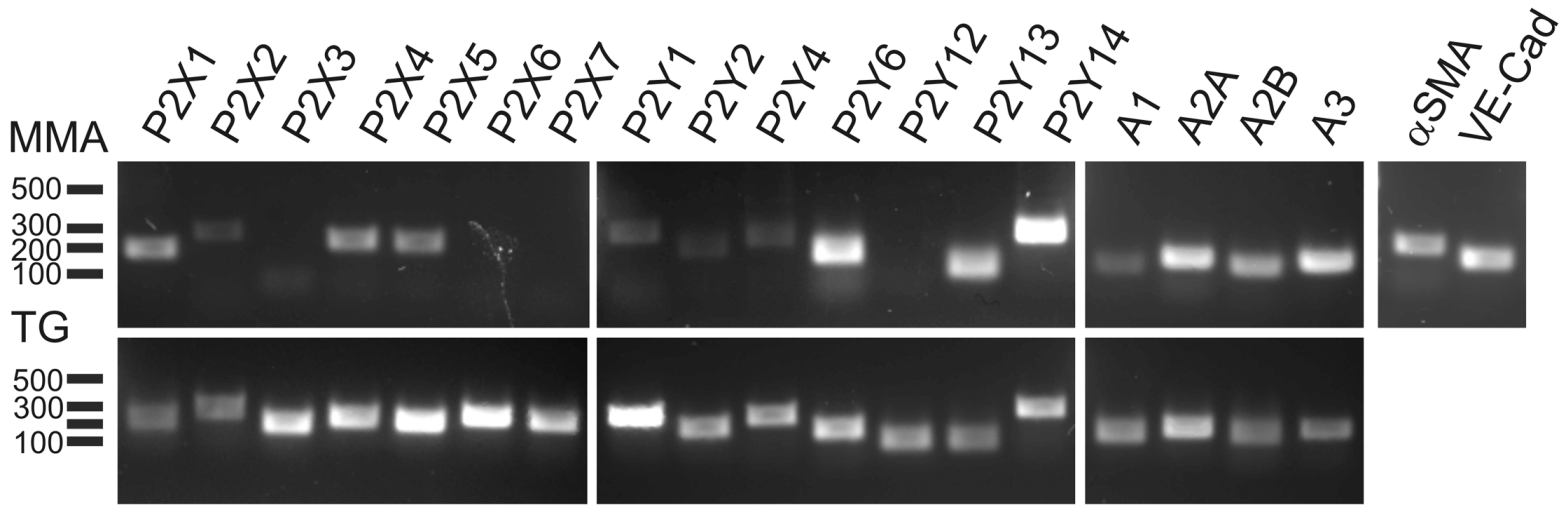
RT-PCR of purinergic receptors in MMA. The top lane is a representative RT-PCR for 3 different runs (each consisting of 6 pooled MMA). The lower lane is a control for all primers run on trigeminal ganglion mRNA. All bands have the correct expected size, see table 1. αSMA (α Smooth Muscle Actin) and VE-Cad (Vascular Endothelial - Cadherin) were used as controls.

In order to further examine the possible purinergic receptor expression in rat MMA, the mRNA transcripts for the different purinergic receptors were studied. The α-smooth muscle actin and VE-Cadherin were used as controls for the presence of smooth muscle cells and endothelial cells, respectively. All data are summarised for 3 individual RNA isolations from 6 pooled animals in each (Table 1) and the PCR (Fig. 7) is representative for the three isolations. Unfortunately, there was not enough material from these small vessels to run qPCR. However, this semi-quantification (Table 1), matches well with the functional data showing clear expression of P2X1, P2Y6/13/14, A2A and A3 receptors. The trigeminal ganglion was used as a positive control for the studies of isolated mRNA and RT-PCR. Interestingly, all purinergic receptor mRNAs are present in the trigeminal ganglion (Fig. 7, lower lane) and at the same base pair size, as for the MMA.

## Discussion

This is the first study to successfully isolate rodent MMAs and study the functional purinergic responses and mRNA expression. In addition an extensive primer database is given, with exon-exon spanning primers that have highly similar Tm. This may allow future fast screening for rat purinergic receptors. We suggest that purinergic regulation is important in MMAs, based on the use of a series of agonists with more or less specificity towards the individual receptors. Interestingly, ADPβS is a strong contractile agonist, which differs from that seen in other arteries [9], [10]. Adenosine causes relaxation of the MMA via a mechanism mainly dependent on A2A receptor that is inhibited by caffeine. Present study gives the first molecular evidence for the long proposed theory on a possible mechanism of adenosine and caffeine in the MMA, putatively involved in headache.

### P2X receptors

The P2X receptors are activated by ATP, however as ATP is rapidly degraded, we probably never reached the full E_max_ with this agonist. We therefore utilised αβmetATP, a stable analogue that is P2X selective [26] (Fig. 3B). The EC_50_ for P2X1 is around 1 µM [27] and we found a pEC_50_ of 6.69±0.18 strongly suggesting that P2X1 receptor is the strong contractile P2X receptor. The P2X1 receptor has also been shown to be the strongest contractile P2X receptor in blood vessels throughout the body [22]. In our expression data, we also observe expressed P2X2 receptor together with P2X4 and P2X5 receptors. P2X2 and P2X4 receptors have a much lower sensitivity to αβmetATP (pEC50>100 µM) [28]. The expression could originate from endothelial cells where it has been shown to be expressed [22]. The expression of the P2X5 receptor is interesting as P2X1/P2X5, P2X2/P2X5, and P2X4/P2X5 heterotrimers have been shown to exist and to modulate the receptor kinetics compared to the homotrimers [29]. However, most humans carry a null-allele for P2X5 [30]. In conclusion we show that the P2X1 receptor is the strongest contractile P2X receptor in the MMA. Interestingly, there are no P2X3 or P2X6 receptors expressed in the MMA. The P2X3 receptor has been shown to be involved in changes (sensitisation) in the trigeminal ganglion, putatively related to migraine pathophysiology [13], [14].

### P2Y receptors

The P2Y receptors are G-protein coupled receptors and have a more long term role, as they desensitise slower and are linked to changes in gene expression. The ADP receptors were studied using the stable ADP analogue, ADPβS. This agonist will stably activate P2Y1, but also the newly discovered P2Y12 and P2Y13 receptors [31]. ADPβS caused strong contraction, with a pEC_50_ of 4.61±0.10 (Fig. 3C). This value is slightly lower than that observed for P2Y1 and P2Y13 receptors [32], [33] and supported by a rather low expression of P2Y1 receptor seen in the RT-PCR (Fig. 7). Interestingly, ADPβS does not induce contractile responses in other arteries e.g. the cerebral, omental or mesenteric arteries in rats [9], [10]. This makes it an interesting observation which could be used as a target for future *in vivo* understanding of the role of purinergic receptors in the MMA.

UTP and UDP resulted in similar E_max_ contractions although UDP had a higher pEC_50_ (Fig. 4A, 6A/B), suggesting that UDP is the strongest contractile agonist. Indeed, when we tested with the non-degradable analogues UTPγS and UDPβS, UDPβS had a significantly higher contraction (Fig. 5C/D). This, together with the observation that the P2Y6 receptor is strongly expressed (Fig. 7), suggests that it is the main pyrimidine receptor. The agonist for P2Y14 receptor, UDP-glucose has recently been shown to cause contraction in mice and porcine arteries [23], [24]. UDP-glucose was added and caused only a small contraction (Fig. 4B). The receptor is strongly expressed, but we cannot rule out that its main function could be something other than contraction, as it is a negative cAMP regulator [34].

All together the functional data show that the most potent contractile agonists in the MMA are αβmetATP(P2X1)≥UDPβS(P2Y6)>>ADPβS(P2Y1/13)>>UTPγS (P2Y2/4) based on EC_50_ (the P2Y14 receptor not included due to the low E_max_). The E_max_ for the strongest P2Y receptor (P2Y6, 184±12% of 60 mM K^+^ response) is higher than for P2X receptors (P2X1 134±9% of 60 mM K^+^ response), but neither of them reach the full level of ET-1 (271±22% of 60 mM K^+^ response) which is here the strongest vasoconstrictor.

### Adenosine and caffeine

There exist 4 adenosine receptors, all of which are expressed in the MMA (Fig. 7), and all of them are sensitive to antagonism by caffeine. The Kd varies slightly between human and rat, but is overall similar [20]. We used 50 µM caffeine, which would be expected to occur in heavy coffee drinkers. According to Denaro and colleagues, ∼3 cups of coffee (3 mg/kg/day) gave 15 µM caffeine in the blood and ∼8 cups (12 mg/kg/day) gave 65 µM [35]. 50 µM therefore corresponds to drinking approximately 6 cups of coffee during the day (100 mg/cup, 70 kg person), which is comparable to acute ingestion of 6 mg/kg, which has been shown to give 45 µM in the blood [36]. All doses of caffeine falls within common caffeine intake, at least in Scandinavian countries where average caffeine consumption is 400 mg/person/day [20].

We assume that A1, A2A and A2B receptors will be completely/partially blocked by 50 µM of caffeine; A3 receptors on the other hand would not (Table 2, adapted from Fredholm [20]). Our data show that the MMAs contract when exposed to adenosine instead of relaxing, in the presence of caffeine (Fig. 6A). This is considered mainly mediated with a blockage of A2A receptor, as the specific antagonist SCH58261 could mimic 60% of the response and also caused adenosine to contract the vessels (Fig. 6B). The difference is likely the sum of A1 and A2B receptors, both being expressed. At the higher adenosine concentrations (>1 µM) we still see a rightward shift of relaxation (Fig. 5A), which is expected as caffeine is a competitive antagonist [16]. However, the blood vessel is always contracted compared to when there is no adenosine present. Our data are the first to show the direct effect of caffeine on dural arteries. Since the Kd values of human and rat are so similar, we postulate that the observations can be extrapolated to humans.

**Table 2:** Kd Values for caffeine antagonism on adenosine receptors.

Receptor subtype	Rat (K_D_) *	Human (K_D_) *
A1 receptor	20 µM	12 µM
A2A receptor	8.1 µM	2.4 µM
A2B receptor	17 µM	13 µM
A3 receptor	190 µM	80 µM

*The Kd values are taken from the review by Fredholm and colleagues [20].

In conclusion, we show that the blood flow through the MMA may at least in part be regulated by purinergic receptors. P2X1 and P2Y6 receptors are the strongest expressed P2 receptors, both functionally and in semi-quantitative PCR. Interestingly, ADPβS causes functional contraction, and receptors for this agonist (P2Y1 and P2Y13) are expressed, suggesting a role in MMA that is not seen in other vessels. Adenosine causes relaxation of MMAs, which is mediated mainly through A2A receptors, the strongest expressed adenosine receptor and is inhibited by SCH58261. This response is also seen with the addition of a physiological caffeine concentration (50 µM), giving for the first time one putative molecular explanation for benefit and use of coffee/caffeine as a MMA vasoconstrictor that could be related to sensation of cranial pain.
